# Changed Caecal Microbiota and Fermentation Contribute to the Beneficial Effects of Early Weaning with Alfalfa Hay, Starter Feed, and Milk Replacer on the Growth and Organ Development of Yak Calves

**DOI:** 10.3390/ani9110921

**Published:** 2019-11-05

**Authors:** Shengru Wu, Zhanhong Cui, Xiaodong Chen, Peiyue Wang, Junhu Yao

**Affiliations:** 1 College of Animal Science and Technology, Northwest A&F University, Xianyang 712100, China; cuizhanhong27@126.com (Z.C.); xiaodongchen2017@nwafu.edu.cn (X.C.); wpy991120@163.com (P.W.); 2 Qinghai Academy of Animal Husbandry and Veterinary Sciences, Qinghai University, Xining 810016, China

**Keywords:** yak calf, early weaning, caecal microbiota, 16S rRNA gene sequencing, growth performance

## Abstract

**Simple Summary:**

Yak calves during the pre-weaning period are mainly fed by maternal grazing and nursing, which is beneficial to the oestrus and mating of female yaks or the survival and growth of calves. Barn feeding and early weaning with mixed rations of available roughage and grains was presented as an alternative to maternal grazing and was supposed to be beneficial to the tremendous ruminal and intestinal development and growth of yak calves. The caecum is also the primary site of microbial fermentation, but the limited research has focused on the role of caecal microbiota in regulating the growth of yaks. The findings of the current study indicated that early weaning by supplying calves with milk replacer, alfalfa hay, and starter feed improves yak calf growth performance compared with maternal grazing and nursing, in part through alterations of caecal microbiota and caecal volatile fatty acid (VFA) production induced by supplementation with alfalfa hay and starter feed.

**Abstract:**

This study aimed to investigate the effect of early weaning by supplying calves with alfalfa hay, starter feed, and milk replacer on caecal bacterial communities and on the growth of pre-weaned yak calves. Ten 30-day-old male yak calves were randomly assigned to 2 groups. The maternal grazing (MG) group was maternally nursed and grazed, and the early weaning (EW) group was supplied milk replacer, starter feed, and alfalfa hay twice per day. Compared with the yak calves in the MG group, the yak calves in the EW group showed significantly increased body weight, body height, body length, and chest girth. When suffering to the potential mechanism of improved growth of yak calves, except for the enhanced ruminal fermentation, the significantly increased total volatile fatty acids, propionate, butyrate, isobutyrate, and valerate in the caecum in the EW group could also serve to promote the growth of calves. By using 16S rDNA sequencing, some significantly increased caecal phylum and genera, which were all related to the enhanced caecal fermentation by utilizing both the fibrous and non-fibrous carbohydrates, were identified in the EW group. In conclusion, early weaning of yak calves by supplying them with alfalfa hay, starter feed, and milk replacer is more beneficial to the growth of yak calves when compared with maternal grazing and nursing, in part due to alterations in caecal microbiota and fermentation.

## 1. Introduction

Yak calves during the pre-weaning period are mainly fed by maternal grazing and nursing, which are not beneficial to the oestrus and mating of female yaks or the survival and growth of calves [1]. However, the pre-weaning period is a critical period for the developmental plasticity and, subsequently, biological function changes of young ruminants [2,3]. Adequate nutrition during early life is beneficial to gastrointestinal microbiota establishment, development, and the subsequent functional transition from metabolizing the glucose from milk to the volatile fatty acids (VFAs) from a solid diet [4,5]. Barn feeding and early weaning with mixed rations of available roughage and grains was presented as an alternative to maternal grazing and was supposed to be beneficial to the tremendous gastrointestinal ramifications and growth of yak calves and other juvenile ruminants [5,6]. In previous studies, the significantly enhanced rumen fermentation and changed rumen microbiota condition were implicated as the main reasons for the observed improved growth performance of cattle and lamb by supplying them with alfalfa hay, starter feed, and milk replacer in barn feeding and early weaning groups [4,5,6], which were rarely studied in the yak calves.

In addition to rumen fermentation, hindgut fermentation, which includes caecal fermentation, is also an important factor that affects growth performance and healthy conditions [4,7]. The caecum is also the major site of fermentation and absorption in the large intestine of ruminants, and approximately 17% of digested cellulose is broken down there [8]. The VFAs produced in the caecum account for 12% of total VFA production in sheep [9]. However, compared with the extensive studies focusing on the rumen microbiota and fermentation, caecal microbiota and fermentation is also an important factor that affects growth performance, which was comparatively limited in studies but worth further studying of the roles of microbiota from different segments in utilizing the nutrients and promote the growth of yak calves. In the present study, the effect of early weaning with alfalfa hay, starter feed, and milk replacer versus maternal grazing and nursing on the caecal microbiota and fermentation of yak calves was evaluated and compared, with the aim of further adding knowledge of changed caecal microbiota in regulating the growth of yak calves. Moreover, we further compared the differences between ruminal and caecal microbiota and fermentation to justify the contribution of caecal microbiota and fermentation on the growth of yak calves.

## 2. Materials and Methods

### 2.1. Ethics Approval Statement

This study was carried out in accordance with the recommendations of the Administration of Affairs Concerning Experimental Animals (Ministry of Science and Technology, China, revised 2004). The protocol was approved by the Institutional Animal Care and Use Committee of the Northwest A&F University (protocol number NWAFAC1118).

### 2.2. Animals, Experimental Design, and Sample Collection

Before the commencement of the trial, all yak calves were only fed with the milk by maternal nursing in Datong Yak Breeding Farm of Qinghai Province. A total of ten 30-day-old male yak calves (34.86 ± 2.06 kg) with similar body conditions were randomly assigned to 2 groups with 5 calves per group. The maternal grazing (MG) group was maternally nursed and grazed, and the early weaning (EW) group was supplied with milk replacer, starter feed, and alfalfa hay. The yak calves in the maternal nursing group had access to fresh grass and yak milk. Briefly, the MG yak calves were allowed to graze a rangeland for a period of 8 h. Water was offered ad libitum twice a day at 08:00 and 16:00 h. Specifically, the experiment was performed from July to October and lasted for 90 d, allowing for the sufficient grazing of fresh grass. Moreover, at the last day of the feeding experiment, the fresh grass and the yak milk were collected and provided to the their yak calves, and the dairy intake were recorded and used to calculate the dry matter intake (DMI). The yak calves in the early weaning group were housed in a barn and kept in individual pens (7 × 4 m). The pens included a sawdust-bedded pack area and a feed lane equipped with an automatic cable scraping system. In addition to free access to starter feed and alfalfa hay, all yak calves in the early weaning group were supplied with milk replacer reconstituted from 100–350 g milk replacer powder (the supplementation of milk replacer were increased along with the increasing body weight) dissolved in 1 L 60 °C water twice per day at 08:00 and 16:30. Water was supplied ad libitum to the yak calves during the experimental period. Feed (include the alfalfa and starter feed) offered was adjusted daily to ensure at least 10% orts. Feed offered and refused by each calf was weighed and recorded on the last day of the feeding experiment. Meanwhile, the daily intake was calculated for further analysis of DMI (overall consideration of the dry matter intake of milk replacer, alfalfa, and starter feed). After the feeding experiment, the yak calves were weighed, and their body size indexes, including the body height, body length, and chest girth, were measured and recorded. Then all animals were euthanized by exsanguination after anaesthesia and immediately dissected, and the liver, thymus, spleen, and pancreas were collected and weighed immediately. At last, the ruminal fluid and caecal contents were collected and stored in −80 °C for further analyses. Specifically, rumen fluid was strained through 4 layers of sterile cheesecloth and collected for VFA and NH3-N analyses and 16S rRNA gene sequencing.

Composites of the fresh grass, starter feed, alfalfa hay, and milk replacer were measured (AOAC International, 2000) for DM (oven method 930.15), ash (oven method 942.05), CP (Kjeldahl method 988.05), fat (alkaline treatment with Röse–Gottlieb method 932.06 for MR; diethyl ether extraction method 2003.05 for starters and hay), Ca and P (dry ashing, acid digestion, and analysis by inductively coupled plasma, method 985.01), NDF with ash without sodium sulfite or α-amylase, ADF with ash, starch (α-amylase method), and sugar (colorimetric method), and the details of the nutrient composition are given in Tables S1 and S2.

### 2.3. Determination Of VFA and NH_3_-N in Ruminal Fluid and Caecal Contents

For the VFA and NH3-N measurements, the rumen fluid and caecal contents dissolved in the buffer were centrifuged at 13,000× *g* for 10 min. The VFAs were analysed on an Agilent 6850 gas chromatograph (Agilent Technologies Inc., Santa Clara, CA, USA) equipped with a polar capillary column (HP-FFAP, 30 m × 0.25 mm × 0.25 μm) and a flame ionization detector (FID), as previously described [10]. The NH3-N in the supernatant was quantified using a continuous-flow analyser (SKALAR San, Skalar Co., Breda, The Netherlands).

### 2.4. Microbial DNA Extraction and 16S rRNA Gene Sequencing

The ruminal fluid and caecal content samples from yak calves were subjected to DNA extraction using the QIAamp DNA Stool Mini Kit (Qiagen, Hilden, Germany). The quantity and quality of those DNA samples were further assessed by a Nanodrop ND-1000 spectrophotometer (Thermo Scientific, Waltham, MA, USA). The 16S rRNA gene amplicons of 8 DNA samples (4 samples from EW group and 4 samples from MG group) with high quality were used to determine the diversity and compare the community structures of the bacterial species in each of these samples using Illumina HiSeq sequencing at Novogene Bioinformatics Technology Co., Ltd., Beijing, China. The preparation of the amplicon library was performed by polymerase chain reaction amplification of the V3–V4 region of the 16S rRNA gene using the primer set 341F 5′-CCTAYGGGRBGCASCAG-3′ and 806R 5′-GGACTACNNGGGTATCTAAT-3′ with barcode. The identified sequences were deposited in the NCBI sequence archive (SRA) under the accession no. PRJNA552771.

Sequencing data splicing and quality filtering of the raw tags were performed using Trimmomatic (V0.36) and Usearch (V9.2.64) [11,12]. All sequences shorter than 200 bp and those with quality scores lower than 15 in the raw reads were removed, and high-quality clean tags were obtained. These sequences were classified into operational taxonomic units (OTUs) at an identity threshold of 97% similarity using UPARSE software [12]. For each OTU, by, a representative sequence was screened and used to assign taxonomic composition by comparison with the RDP 16S Training set (v16) and the core set using the SINTA (Usearch V9.2.64) and PyNAST (QIIME) programmed algorithms [13,14]. Subsequent analysis of alpha and beta diversity was performed based on the output of this normalized data. The taxon abundance for each sample was determined according to phylum, class, order, family, and genus. The *t*-test was performed to estimate the differential microbiota between the treatments. The threshold was set at *p* value < 0.05.

### 2.5. Statistical Analysis

Analysis was performed using Student’s *t* test with SPSS 21.0 software with replicates as experiment units, and differences were considered statistically significant at *p* < 0.05.

## 3. Results and Discussion

### 3.1. Early Weaning of Yak Calves with Alfalfa Hay, Starter Feed, and Milk Replacer Significantly Promoted Growth and Organ Development

Compared with the yaks in the maternal grazing group, the yaks in the early weaning group showed significantly increased body weight, withers height, body length, and chest girth (Table 1). Additionally, the significantly increased weight of the liver, spleen, and thymus, as well as the significantly increased indexes of spleen and thymus (g/kg body weight) were also identified in the early weaning group (Table 1). Meanwhile, the ruminal fermentation characteristics of yak calves under the grazing and barn feeding conditions are presented in Table 2. The PH and NH3-N showed no differences between the different feeding groups. The total VFA concentration was significantly higher in the early weaning group than in the grazing group; of these, the propionate, butyrate, isobutyrate, and valerate were also significantly increased in the early weaning group (Table 2). Furthermore, the ratio of acetate/propionate and acetate/total VFA were significantly decreased in the early weaning group, while the ratio of butyrate/total VFA, isobutyrate/total VFA, and valerate/total VFA were all significantly increased in the early weaning group. Moreover, those significantly increased growth performance and ruminal fermentation were mostly resulted from the significant differences between the treatments in the daily DMI of yak calves, where the increased intake was found for calves on early weaning group, especially the increased intakes of concentrate supplement (Table 1).

Our results indicated that the early weaning yak calves provided with milk replacer, starter feed, and alfalfa hay during early life showed improved growth and development, in accordance with the results of previous studies on lambs during early life [5,6]. Supplementation of the diets of ruminants with carbohydrates such as alfalfa hay and starter feed during the pre-weaning period has a crucial long-term impact on ruminal fermentation in other ruminants that has been shown to be beneficial to their growth performance [15]. In accordance with the previous studies, DMI and ruminal VFA production were both significantly increased, which contributed to the significantly promoted growth performance of yak calves in early weaning group [5,6,10,15]. However, except for the VFAs from ruminal fermentation, the caecum VFAs produced accounted for 12% of total VFA production in sheep [9,16], while limited research focused on caecal fermentation in response to the early weaning with starter feed and alfalfa hay in yaks [7].

### 3.2. Significantly Enhanced Caecel Fermentation Was Identified in the Yak Calves in the Early Weaning Group

The caecal fermentation characteristics of yak calves under the grazing and early weaning conditions were further measured (Table 2). The pH and NH_3_-N also showed no differences between the two groups. The total VFA concentration was significantly higher in the early weaning group than in the grazing group (*p* = 0.026). Significantly higher concentrations of propionate, butyrate, and other VFAs were also identified in the early weaning group (*p* < 0.01), whereas concentration of acetate was not significantly altered by treatment in the present study. Meanwhile, the ratio of acetate/propionate and acetate/total VFA in caecum were also significantly decreased in the early weaning group, while the ratio of butyrate/total VFA and propionate/total VFA were both significantly increased in the early weaning group. Moreover, according to our results, we found that the concentration and production of VFAs in the caecum could not be ignored when compared with the ruminal VFA concentration. In ruminants including the yak calves, the VFAs are absorbed by the rumen and caecal epithelium, and then metabolized into glucose, triglycerides, and amino acids and further provide energy and nutrients resources for the growth of yak calves. Our results indicated that changed caecal VFAs in early weaning, especially the increased acetate and propionate induced by supplementation of the diet with alfalfa hay and starter feed, can also be absorbed by the caecal epithelium [5,17], and then metabolized into glucose, triglycerides, and amino acids and further provide energy and nutrients resources for the growth of yak calves [7]. Moreover, the increasing ratio of butyrate/total VFA and propionate/total VFA further represented the improved energy utilization efficiency when compared with the acetate type fermentation and the promoted caecal development process.

### 3.3. Different Responses of Caecal Microbiota to Early Weaning or Maternal Grazing Feeding Contribute to Enhanced Caecal Fermentation of Yak Calves

**Figure 1:**
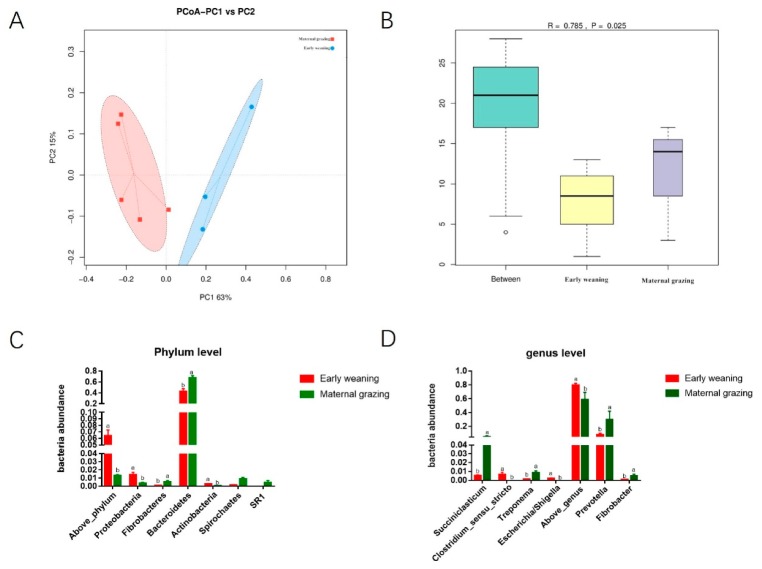
Ruminal microbial community difference between the different feeding paradigm groups (n = 4). (**A**) PCoA analysis. (**B**) Anosium analysis. (**C**) Differential ruminal microbes at phylum level based on *t*-test analysis. (**D**) Differential ruminal genera based on *t*-test analysis.

**Figure 2:**
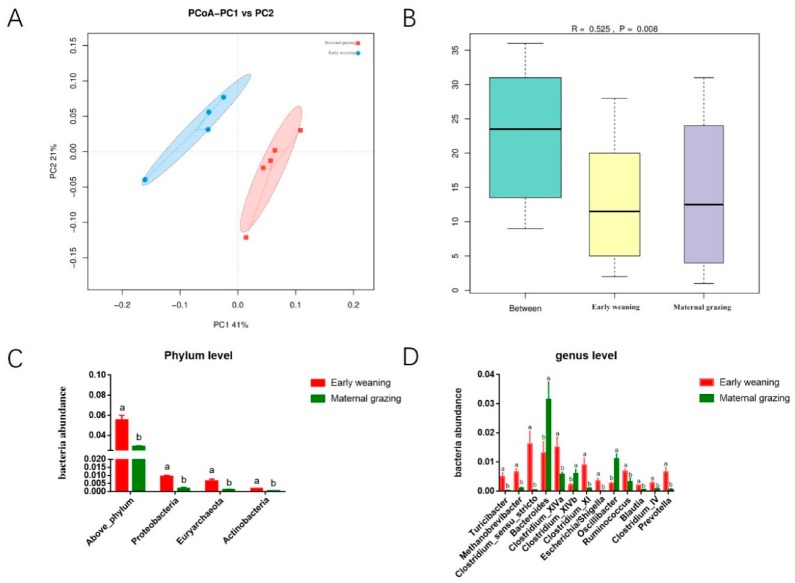
Caecal microbial community difference between the different feeding paradigm groups (n = 4). (**A**) PCoA analysis. (**B**) Anosium analysis. (**C**) Differential caecal microbes at phylum level based on *t*-test analysis. (**D**) Differential caecal genera based on *t*-test analysis.

Considering the significantly increased ruminal and caecal VFAs, the ruminal and caecal microbiota were both further analysed. The beta diversity analyses revealed that the compositions of the gastrointestinal prokaryotic community of the yak calves in two different feeding groups were significantly different (Figure 1A,B, and Figure 2A,B). Moreover, Chao1 indexes indicated that early weaning with the starter feed and alfalfa hay was beneficial to the diversity of ruminal and caecal microbiota (Table S3). Recently, several studies have focused on the effect of different feeding paradigms on the gastrointestinal microbiota of several animals, including lizards, cheetahs, yaks, lambs, deer mice, and seals [18,19,20,21]. These studies all identified that the diversity and abundance of the gastrointestinal microbiota were increased in animals from the wild environment than in captive animals. However, our study identified that early weaning and barn feeding significantly increased the diversity of ruminal and caecel microbiota. Different from the previous studies which focused on adult animals which obtained more varied nutrients in wild feeding paradigms, the early weaning in the barn feeding paradigm in the present study, which supplied the calves with milk replacer, starter feed, and alfalfa hay, provided enough carbohydrate, protein, and lipid for the growth and proliferation of microbes, which indicated that more microbial species could survive in the gastrointestinal tract due to the abundant sources of carbon and nitrogen [7,17]. In contrast, the yak calves from the maternal grazing group obtained limited nutrients from maternal milk and fresh grass, which resulted in less diversity and abundance of the ruminal and caecal microbiota. Moreover, the similar results of the increased diversity of ruminal and caecal microbiota also indicated that the dietary changes can simultaneously altered the ruminal and caecal microbiota, which also indicated that caecal microbiota could also have potential response to dietary supplementation and further influence the growth of yak calves. Overall, our results indicated that the nutrition supplementation is beneficial to the richness of caecal microbiota such as the alteration of the diversity of ruminal microbiota, which could even changeover the beneficial effect of a wild environment on the diversity and richness of gut microbiota. Our results also indicated that nutrition was the main effect among environmental indices which influence the gastrointestinal microbiota [22].

The differential microbiota were further identified based on the counts of different microbes by using *t*-test analyses. In rumen, the significantly increased phylum of Proteobacteria, Fibrobacteres, Bacteroidetes, Actinobacteria, Spirochaetes, and SR1 (Figure 1C), as well as the genus of Succiniclasticum, Clostridium_sensu_stricto, Treponema, Escherichia/Shigella, Prevotella, and Fibrobacter (Figure 1D) were identified in the early weaning group. In caecum, the significantly increased Proteobacteria, Euryarchaeota, and Actinobacteria in the phylum level (Figure 2C), as well as significantly increased genera of Turicibacter, Methanobrevibacter, Clostridium_sensu_stricto, Bacteroides, Clostridium_XlVb, Clostridium_XI, Escherichia/Shigella, Oscillibacter, Ruminococcus, Blautia, Clostridium_IV, and Prevotella (Figure 2D) were identified in the early weaning group. Accordingly, the phylum of Proteobacteria, Euryarchaeota, and Actinobacteria and the genera of Clostridium_sensu_stricto, Escherichia/Shigella, and Prevotella were co-influenced by the early weaning with alfalfa hay, starter feed, and milk replacer, which were all involved in the utilization of fibrous and non-fibrous carbohydrates and the production of propionate and butyrate. Meanwhile, these results again proved that dietary alteration could have a similar effect on the ruminal and caecal microbiota. Moreover, the main finding of our study lies in the fact that supplemental feeding with alfalfa hay and starter feed exceeded maternal grazing and nursing in shaping hindgut functional achievement. The significantly increased caecal genera of yak calves identified in the early weaning groups, including the *Prevotella*, *Clostridium_XIVb*, *Turicibacter*, *Clostridium_IV*, *Clostridium_XI*, *Clostridium_sensu_stricto*, *Bacteroides*, *Oscillibacter* and, *Ruminococcus* were mainly involved in the utilization of fibrous and non-fibrous carbohydrates and the production of acetate, propionate, and butyrate [17,23,24,25,26,27]. The primary determinant for this could be that the early-weaning calves consumed a greater amount of concentrate and alfalfa hay, and dietary fiber and starch were the suitable fermentation substrate when they reached the hindgut in significant quantities. Considering the identified significantly increased VFAs in the present study, the effect of early weaning with alfalfa hay and starter feeds on the identified variational microbiota and the roles of these changed microbiota were again proved. According to previous studies, in goats during the early life, caecal propionate, butyrate, and isobutyrate concentrations also significantly increased in response to a grain-rich diet [7,17,28]. In accordance with these previous studies, significantly higher concentrations of propionate, butyrate and total VFAs were also identified in the early weaning group of the present study, which were produced by our identified differential microbes by using starch or fibrous carbohydrates. Moreover, the effect of differential supplementing of carbohydrates during early life, induced by early weaning with alfalfa hay and starter feed, on the subsequent gastrointestinal microbiota and the related caecal fermentation, could further increase the absorbed VFAs from the caecal epithelium, and further provide more energy for the growth of yak calves [29,30]. Overall, except for ruminal fermentation, caecal fermentation could also be enhanced by providing enough fermentable carbohydrates in the EW group, which was induced by the increased abundance of microbes involved in the utilization of fibrous and non-fibrous carbohydrates and subsequently increased; and then the increased caecal VFAs could contribute to promoting the growth of yak calves.

## 4. Conclusions

Early weaning and barn feeding with milk replacer, alfalfa hay, and starter feed is recommended during pre-weaning to improve yak calf growth performance. Except for their beneficial roles in ruminal microbiota construction and ruminal VFAs production, the facilitating caecal starch-using and fibre-using microbial colonization and the subsequently improved caecal fermentation can also contribute to the growth of yak calves, which may play similar roles to the changed ruminal microbiota.

## Figures and Tables

**Table 1:** Effect of early-weaning feeding and maternal grazing feeding on body weight, body size indexes, and organ weight of yak calves.

Items	Treatments		SEM	*p*-Value
	Early Weaning	Maternal Grazing		
Body weight (kg)	87.90 ^a^	64.50 ^b^	4.347	<0.001
Chest girth (cm)	116.20 ^a^	107.25 ^b^	1.956	0.009
Withers height (cm)	95.60 ^a^	77.25 ^b^	31.563	0.001
Body length (cm)	109.20 ^a^	86.00 ^b^	4.215	<0.001
Liver (g)	1391.50 ^a^	1058.38 ^b^	61.067	<0.001
Spleen (g)	237.84 ^a^	150.60 ^b^	17.554	0.002
Thymus (g)	252.96 ^a^	105.25 ^b^	26.176	<0.001
Pancreas (g)	50.56	50.85	1.694	0.939
Liver index (g/kg body weight)	1.584	1.605	0.023	0.677
Spleen index (g/kg body weight)	0.272 ^a^	0.228 ^b^	0.011	0.050
Thymus index (g/kg body weight)	0.288 ^a^	0.160 ^b^	0.023	<0.001
Pancreas index (g/kg body weight)	0.057 ^b^	0.077 ^a^	0.039	0.002
DMI (g)	1774.60	1147.52	21.303	<0.001

^a,b^ within a row with different superscripts means significantly difference.

**Table 2:** Effect of early-weaning feeding and maternal grazing feeding on caecal fermentation of yak calves.

Items		Treatments		SEM	*p*-Value
		Early Weaning	Maternal Grazing		
Rumen	pH	6.88	7.17	0.128	0.298
	Ammonia nitrogen, NH_3_N (mg/dL)	6.91	6.55	0.234	0.486
	Total VFA (mmol/L)	66.82 ^a^	58.00 ^b^	1.767	0.002
	Acetate (mmol/L)	42.09	40.95	0.758	0.493
	Propionate (mmol/L)	11.53 ^a^	9.85 ^b^	0.346	0.004
	Butyrate (mmol/L)	8.60 ^a^	4.08 ^b^	0.801	<0.001
	Isobutyrate (mmol/L)	1.56 ^a^	1.03 ^b^	0.117	0.009
	Valerate (mmol/L)	1.18 ^a^	0.55 ^b^	0.118	<0.001
	Isovalerate (mmol/L)	1.87	1.54	0.086	0.058
	Acetate/Propionate	3.66 ^b^	4.16 ^a^	0.111	0.011
	Acetate/Total VFA	0.630 ^b^	0.706 ^a^	0.014	<0.001
	Propionate/Total VFA	0.173	0.170	0.002	0.580
	Butyrate/Total VFA	0.129 ^a^	0.071 ^b^	0.010	<0.001
	Isobutyrate/Total VFA	0.023 ^a^	0.018 ^b^	0.001	0.035
	Valerate/Total VFA	0.018 ^a^	0.010 ^b^	0.002	<0.001
	Isovalerate/Total VFA	0.0279	0.0265	0.001	0.482
cecum	pH	6.92	6.95	0.033	0.736
	Ammonia nitrogen, NH_3_N(mg/dL)	6.86	6.80	0.177	0.874
	Total VFA (mmol/L)	63.53 ^a^	56.37 ^b^	1.601	0.012
	Acetate (mmol/L)	41.45	40.52	0.861	0.624
	Propionate (mmol/L)	11.93 ^a^	9.60 ^b^	0.427	<0.001
	Butyrate (mmol/L)	5.51 ^a^	3.08 ^b^	0.465	<0.001
	Other (mmol/L)	4.64 ^a^	3.17 ^b^	0.306	0.004
	Acetate/Propionate	3.48 ^b^	4.22 ^a^	0.148	0.002
	Acetate/Total VFA	0.652 ^b^	0.719 ^a^	0.012	0.001
	Propionate/Total VFA	0.188 ^a^	0.171 ^b^	0.004	0.024
	Butyrate/Total VFA	0.087 ^a^	0.055 ^b^	0.006	0.002

^a,b^ within a row with different superscripts means significantly difference.

## Supplementary Materials

The following are available online at https://www.mdpi.com/2076-2615/9/11/921/s1, Table S1: Nutrient composition of the alfalfa, starter feed, and milk replacement used in the present study; Table S2. Nutrient content of fresh grass and yak milk for yak calves from maternal grazing group; Table S3 Effect of early-weaning feeding and maternal grazing feeding on caecal microbial alpha diversity index of yak calves.
