# Supplementary material for: Changed Caecal Microbiota and Fermentation Contribute to the Beneficial Effects of Early Weaning with Alfalfa Hay, Starter Feed, and Milk Replacer on the Growth and Organ Development of Yak Calves
Source: Animals (Basel). 2019 Nov 5;9(11):921. doi: 10.3390/ani9110921 (PMC6912816; doi:10.3390/ani9110921)
Supplement: Supplementary file 1 [file animals-09-00921-s001.zip › Supporting information.docx]

**Title:** Changed caecal microbiota and fermentation contribute to the beneficial effects of early weaning with alfalfa hay, starter feed, and milk replacer on the growth and organ development of yak calves

**Authors:** Shengru Wu^*,1^, Xiaodong Chen^*^, Zhanhong Cui^*, #^, Peiyue Wang*, Junhu Yao^*, 1^

**Institution:** * College of Animal Science and Technology, Northwest A&F University, Yangling, Shaanxi, China

# Qinghai Academy of Animal Husbandry and Veterinary Sciences, Qinghai University, Xining, Qinghai, China

**^1^ Corresponding author:**

Shengru Wu: [wushengru2013@163.com](mailto:wushengru2013@163.com), [wushengru2013@nwafu.edu.cn](mailto:wushengru2013@nwafu.edu.cn);

Junhu Yao, email: [yaojunhu2004@sohu.com](mailto:yaojunhu2004@sohu.com), [yaojunhu2008@nwsuaf.edu.cn](mailto:yaojunhu2008@nwsuaf.edu.cn); Fax: +86 29 87092164; Tel.: +86 13891883031.

**Supporting information**

**Table S1. Nutrient composition of the alfalfa, starter feed, and milk replacement provided to the yak calves from barn feeding group in the present study.**

**Table S2. Nutrient content of fresh grass and yak milk for yak calves from maternal grazing group.**

**Table S3. Effect of early-weaning feeding and maternal grazing feeding on caecal microbial alpha diversity index of yak calves**

**Table S1. Nutrient composition of the alfalfa, starter feed, and milk replacement used in the present study.**

| Items (%) | milk replacer | alfalfa | starter feed |
| --- | --- | --- | --- |
| Dry matter (DM, % as-fed ) | 94.00 | 93.80 | 91.0 |
| Sugar (% of DM) | - | - | 6.5 |
| Starch (% of DM) | - | - | 40.5 |
| Crude protein (CP, % of DM) | 24.00 | 12.50 | 19.70 |
| Ether extract (EE, % of DM) | 16.00 | 0.90 | 3.80 |
| Neutral detergent fiber (NDF, % of DM) | - | 56.45 | 10.90 |
| Acid detergent fiber (ADF, % of DM) | - | 40.40 | 4.10 |
| Calcium (Ca, % of DM) | 0.60～3.00 | 9.81 | 14.07 |
| Phosphorus (P, % of DM) | 0.50～2.00 | 0.18 | 0.61 |
| Lysine (% of DM) | 2.20 | 0.85 | 1.14 |
| Methionine (% of DM) | - | 1.73 | 1.94 |

**Table S2. Nutrient content of fresh grass and yak milk for yak calves from maternal grazing group.**

| Pasture | | Yak milk | |
| --- | --- | --- | --- |
| Wet pasture production（g/m^2^） | 168.13 | Milk production（kg/d） | 1.04 |
| Dry matter (DM)（%） | 53.24 | Total solid constituent（%） | 17.45 |
| Crude protein (CP)（%） | 10.48 | Milk fat（%） | 6.58 |
| Ether extract (EE)（%） | 2.82 | Milk protein（%） | 5.03 |
| Neutral detergent fiber (NDF)（%） | 49.03 | Lactose（%） | 4.93 |
| Acid detergent fiber (ADF)（%） | 29.53 |  |  |
| Ash（%） | 8.48 |  |  |

**Table S3** **Effect of early-weaning feeding and maternal grazing feeding on caecal microbial alpha diversity index of yak calves**

| Items | Treatmens | | SEM | *P*-value |
| --- | --- | --- | --- | --- |
|  | Early weaning | Maternal grazing |  |  |
| Chao1 | 1135.50^a^ | 627.90^b^ | 66.272 | ＜0.001 |
| Shannon_2 | 6.99 | 6.69 | 6.672 | 0.420 |
